# Supplementary material for: Abscisic Acid Enhances Trehalose Content via OsTPP3 to Improve Salt Tolerance in Rice Seedlings
Source: Plants (Basel). 2023 Jul 17;12(14):2665. doi: 10.3390/plants12142665 (PMC10383865; doi:10.3390/plants12142665)
Supplement: Supplementary file 1 [file plants-12-02665-s001.zip › plants-2473715-supplementary.pdf]

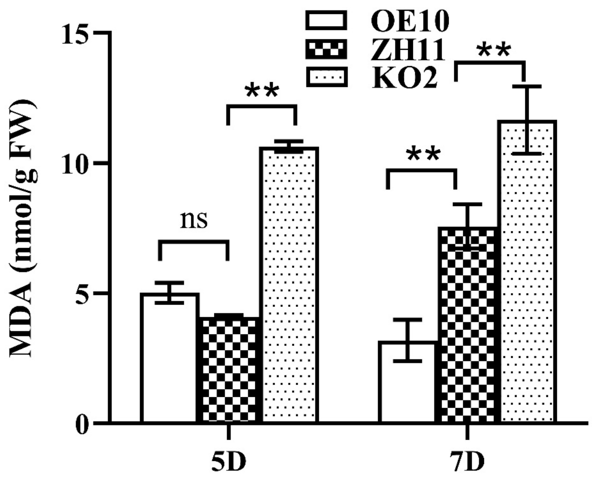

Figure S1 MDA content in OE10, KO2 and ZH11 seedlings under salt stress. 8-days-old seedlings of OE10, KO2 and ZH11 were treated with 100mM NaCl. Shoot were sampled at 5d and 7d after salt stress for MDA content analysis. Vertical bar indicates the mean of three replicates ±SD. One-way analysis of variance was used to do the comparisons, $**P < 0.001$ was considered as statistically significant, ns means not significant.
